# Role of high-resolution computerized tomography chest in identifying tubercular etiology in patients diagnosed as Eales’ disease

**DOI:** 10.1186/s12348-016-0120-1

**Published:** 2017-01-18

**Authors:** Ranju Kharel (Sitaula), Vandana Iyer, Veena Noronha, Parthopratim Dutta Majumder, Jyotirmay Biswas

**Affiliations:** 1 Department of Ophthalmology, B. P. Koirala Lions Centre for Ophthalmic Studies, Tribhuvan University, Institute of Medicine, Kathmandu, Nepal; 2 Medical Research Foundations, Sankara Nethralaya, 18- College Road, 600006 Chennai, India; 3 Department of Uvea & Intraocular Inflammation, Sankara Nethralaya, Chennai, India; 4 VRR Scan, Chennai, India; 5 Director of Uvea and Ocular Pathology, Medical and Vision Research Foundations, Sankara Nethralaya, 18- College Road, 600006 Chennai, India

**Keywords:** HRCT chest, Eales’ disease, Vasculitis, Tuberculosis

## Abstract

**Background:**

The high resolution computerized tomography of chest is an important diagnostic imaging tool to identify any pulmonary tubercular lesion. It's role in Eales' disease to identify any possible association with pulomonary tuberculosis has not been studied earlier. So, this study was conducted to assess the role of high resolution computerized tomography (HRCT) chest in identifying tuberculous etiology in Eales' disease.

**Results:**

It was a retrospective study conducted at a tertiary care eye hospital in South India between January 2009 and October 2014 were included. A total of 29 diagnosed cases of Eales' (24 male and 5 female) were included in the study. These patients were followed up for a mean period of 739.75 days. Out of them, 13 (44.8%) had bilateral and 16 (55.2%) had unilateral ocular involvement. Eight cases (34.5%) patients had vitreous inflammation. Mantoux test was positive in 12 (41.4%) cases and chest x-ray suggestive of TB was present in four cases (13.8%). QuantiFERON TB gold was positive in 15 (51.7%) and HRCT chest suggestive of TB was positive in 15 (51.7%) case. Out of 15 Eales' cases with positive HRCT scan suggestive of TB, the commonly noted lesions were calcified nodules 34.5%, mediastinal hilar lymphadenopathy 13.8%, parenchymal soft tissue lesions in 3.4%.

Five (17.2%) cases underwent pars plana vitrectomy for non resolving vitreous hemorrhage and one case underwent retinal attachment surgery with encirclage. Six patients were started on 9 months regimen of ATT by the chest physician. Final visual outcome improved in 17(40.5%) eyes, maintained in 21(50%) eyes but vision deterioration in 7(16.7%) eyes.

**Conclusions:**

HRCT chest is an important diagnostic tool to rule out pulmonary tuberculosis in Eales’ disease.

## Background

Eales' disease is an idiopathic inflammatory venous occlusion of the peripheral retina of young adults. It is characterized by periphlebitis, peripheral capillary nonperfusion, and neovascularizations [1, 2]. The most common presentation is bilateral recurrent vitreous hemorrhage and its sequelae [3]. Eales' disease is usually a diagnosis of exclusion [4]. Several systemic diseases have been thought to be associated with its occurrence but none of the associations have been proven. The association of *Mycobacterium tuberculosis* as a causative agent in Eales' is still not well understood. Cell mediated immunity against tubercular antigen was reported to be associated with Eales' disease [5]. Polymerase chain reaction (PCR) studies on vitreous sample and epiretinal membrane of Eales' disease patients have confirmed the presence of Mycobacterium genome [6, 7]. Confirmation of tubercular cause in a case of Eales' disease helps in starting evidence based and target oriented therapy for Eales' disease thereby leading to a better resolution of the disease and less number of recurrences.

HRCT chest is an efficient non-invasive diagnostic tool which can differentiate between active and inactive form of tuberculosis (TB), can identify post primary TB, and can demonstrate pulmonary cavity lesions, endobronchial lesions and mediastinal hilar lymphadenopathy [8]. It is also of immense help in differentiation of similar granulomatous conditions. The use of HRCT chest for the detection of tuberculosis in Eales' disease has not been studied so far. In this study, we evaluated the utility of HRCT chest for the detection of tubercular lesions in lungs of Eales' patients in a TB-endemic country like India.

## Methods

This study was a 5-year retrospective hospital-based analysis of patients of Eales' disease presenting to a tertiary care eye hospital in south India, between January 2009 and October 2014.

All patients with clinical diagnosis of Eales' disease were included in the study. Eales' disease was diagnosed based on the features of midperipheral retinal periphlebitis, peripheral capillary non-perfusion, neovascularizations, and recurrent vitreous hemorrhage in healthy young males in the absence of any other possible systemic association [5].

Detailed ocular examination was performed. Fundus fluorescein angiogram (FFA) was done in all cases to rule out capillary non-perfusion area and neovascularization at disc and elsewhere. The staging was done using new staging system for idiopathic retinal periphlebitis as described by Saxena and Kumar [9]. Tuberculin skin test with five PPD, serum angiotensin enzyme levels, vasculitis work-up, chest X-ray, QuantiFERON TB Gold test, and HRCT chest were done. For the HRCT chest, images of 1-mm thickness were taken at 30 mm spacing from lung apices to lung bases, at the end of full inspiration. All the scans were studied for lung parenchyma, mediastinum, pleura, and chest wall. A high-resolution (bone) algorithm was used for image reconstruction. The plain HRCT was routinely performed, and contrast scan was done as per requirement. Single radiologist by a single blinding technique recorded the pattern of chest findings, analyzed and grouped into four groups according to the classification by Ganesh et al. [10]. The decision for antitubercular treatment (ATT) was taken by a chest physician after correlating the clinical findings with HRCT. The patients with at least 2-year follow-up were included in the analysis and recurrences during this period analysed.

Approval from the ethical committee of the institution was taken, and adherence to the tenets of Declaration of Helsinki was maintained. Statistical analysis was performed using Statistical software (SPSS 14). Confidence intervals of 95% was taken, and *p* value <0.05 was considered to be significant. A *Z* test of hypothesis for proportion was used, and a two-tail *t* test was used to compare sensitivity values.

## Results

A total of 42 eyes of 29 Eales' patients were included in the study; among them, 24 (82.7%) were male and five (17.3%) were female. The average age at presentation was 32.58 ± 7.4 years (range 16–46 years). Bilateral ocular involvement was present in 13 (44.8%) eyes and unilateral in 16 (55.2%) eyes. Involvement of the left eye was found in 17 (40.5%) and the right eye in 25 (59.5%). The mean duration of follow-up was 739.75 days.

**Fig. 1:**
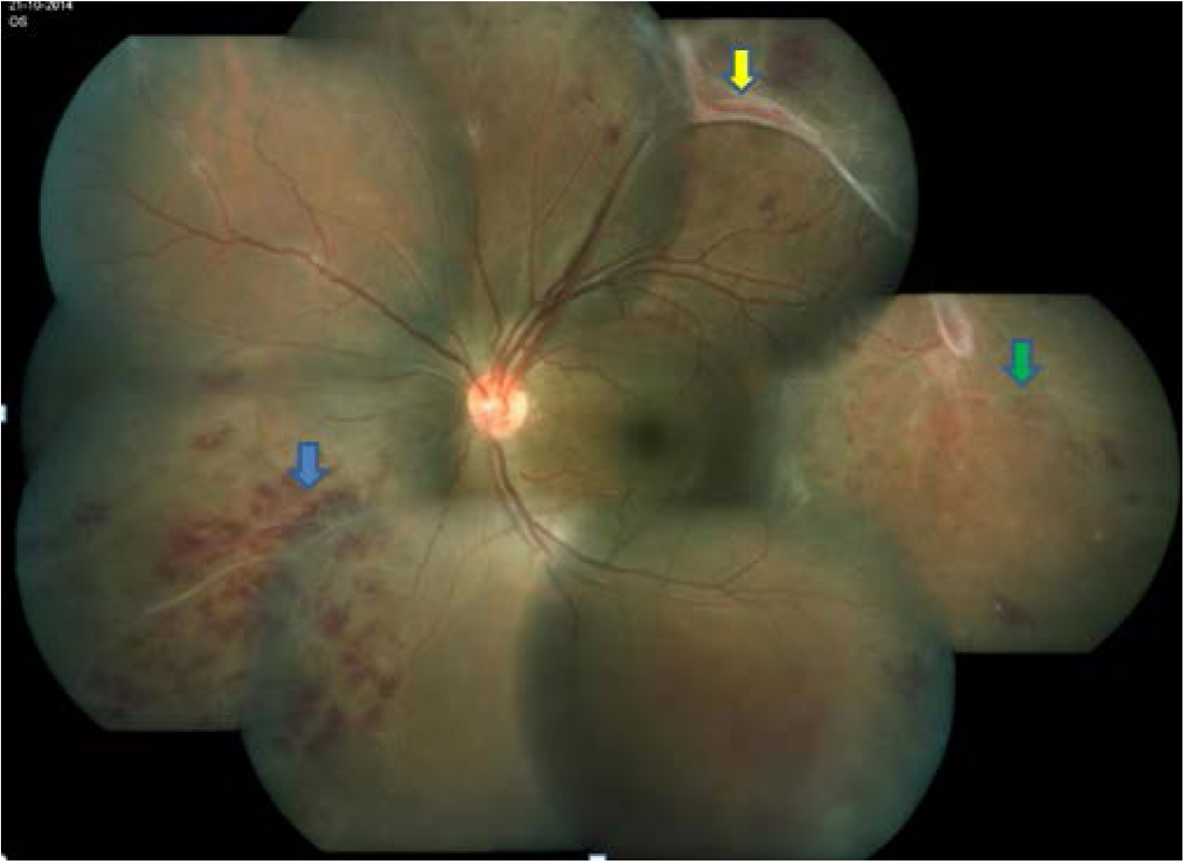
Montage fundus photograph of an Eales' patient showing peripheral retinal hemorrhage (*blue arrow*), sclerosed vessels (*green arrow*), and fibrovascular traction (*yellow arrow*)

The commonest slit lamp finding was the presence of vitreous cells in eight cases (19%). No patients had cellular reaction in anterior chamber. The commonest fundus finding was midperipheral phlebitis in 73.3% (Fig. 1) followed by vitreous hemorrhage (22.2%) and tractional retinal detachment (4.4%). There were three eyes (6.6%) with features of venous occlusion. The stage of presentation of Eales' is mentioned in Table 1.

**Table 1:** Stages of the Eales' cases

Stage	Number	Percentage
Stage 1a	4	9.5
Stage 1b	21	50
Stage 2a	1	2.4
Stage 2b	2	4.8
Stage 3a	4	9.5
Stage 3b	10	23.8
Stage 4a	2	4.8
Stage 4b	1	2.4
Total affected eyes	42	100

Mantoux test was positive in 12 (41.4%) cases, and chest X-ray suggestive of TB was present in four cases (13.8%). QuantiFERON TB Gold was positive in 15 (51.7%), and HRCT chest suggestive of TB was positive in 15 (51.7%) cases (Table 2).

**Table 2:** Results of various tests done for work up of tuberculosis in Eales' patients

Investigation	Positive	Negative	Total
Chest X-ray	4 (13.8%)	25 (86.2%)	29 (100%)
Mantoux test	12 (41.4%)	17 (58.6%)	29 (100%)
QuantiFERON TB Gold	15 (51.7%)	14 (48.3%)	29 (100%)
HRCT chest	15 (51.7%)	14 (48.3%)	29 (100%)

Among the various investigations performed, six Eales' cases (20.7%) had positive Mantoux test along with QuantiFERON TB Gold and HRCT chest scan for tuberculosis (Table 3).

**Table 3:** Comparison between various diagnostic tests

Investigations	Number	Percentage
Mantoux test + QuantiFERON TB Gold test + HRCT chest scan positive	6	20.7
Mantoux test + QuantiFERON TB Gold test positive	9	31
Mantoux test + HRCT chest positive	10	35.5
HRCT + QuantiFERON TB Gold positive	10	35.5

**Fig. 2:**
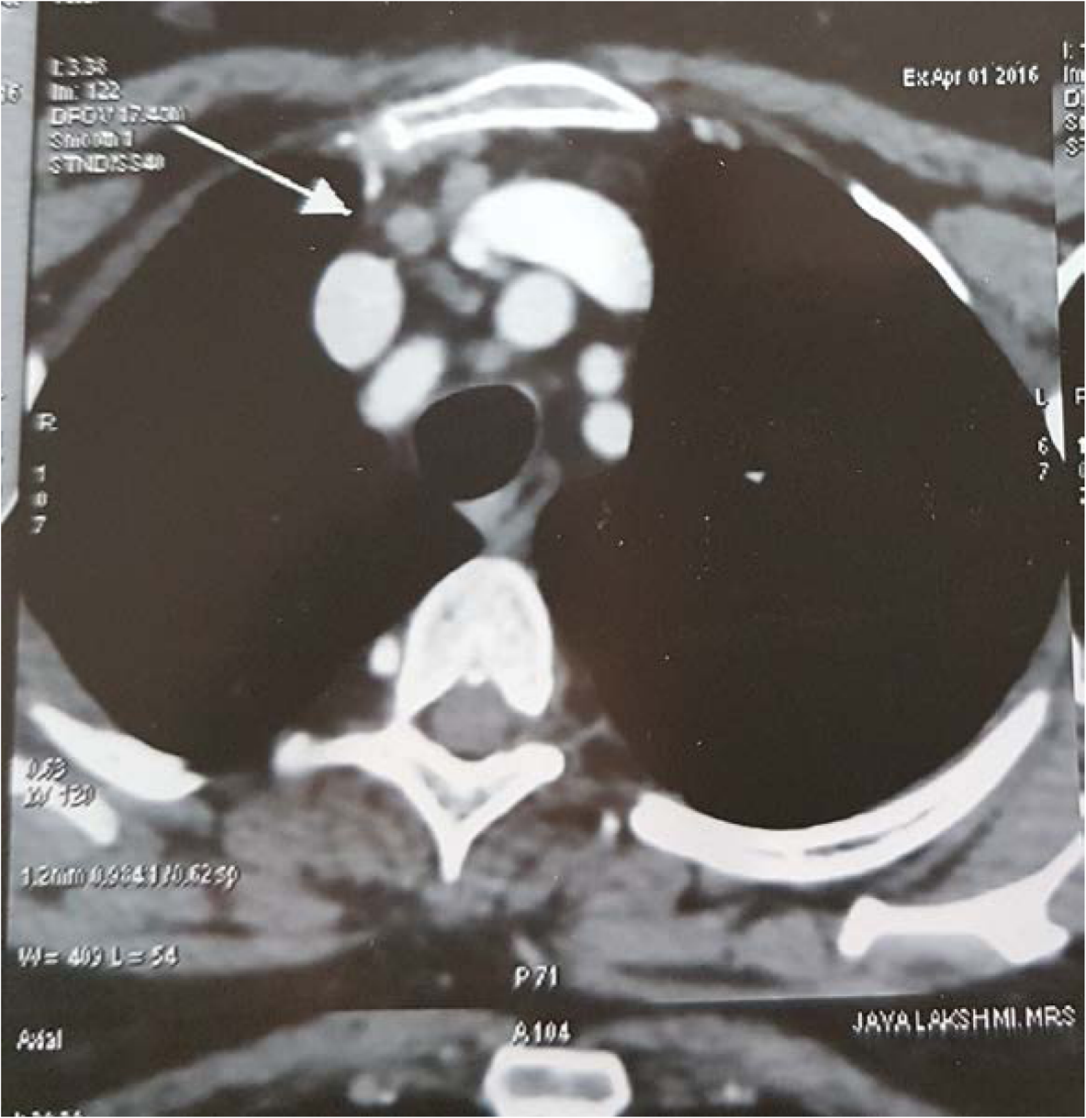
Plain HRCT chest showing calcified and non-calcified lung nodules

**Fig. 3:**
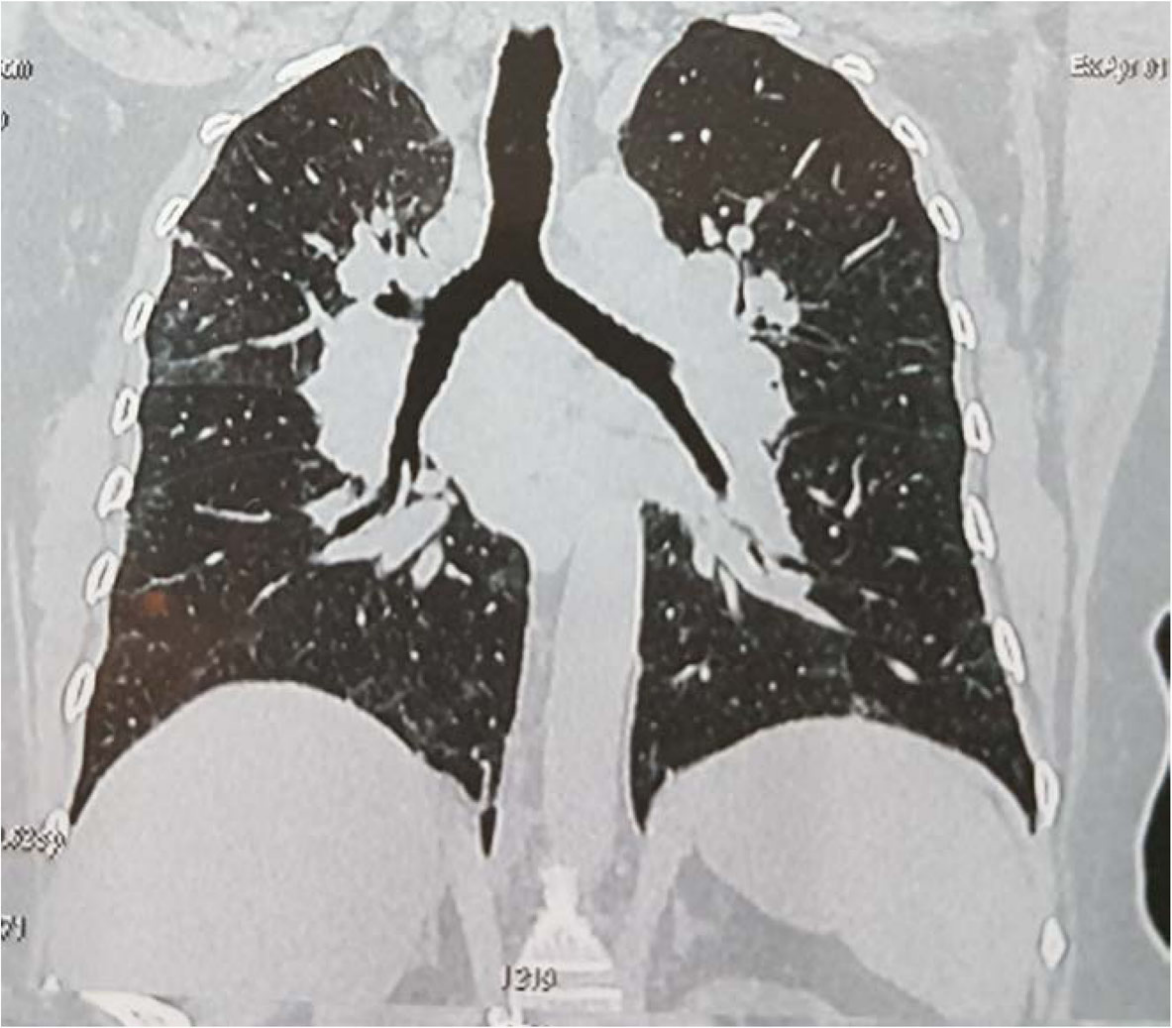
Coronal section of HRCT chest of Eales' showing tree bud appearance of the enlarged hilar lymph nodes

**Fig. 4:**
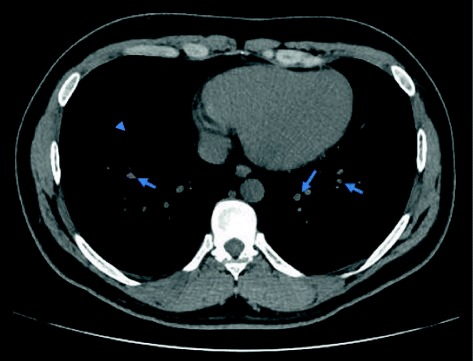
Plain HRCT chest depicting parenchymal lesion with calcification

Out of the 15 HRCT chest scans suggestive of TB, the lesions commonly noted on HRCT were calcified nodules (34.5%) (Fig. 2), mediastinal hilar lymphadenopathy (13.8%) (Fig. 3), parenchymal soft tissue lesions in (3.4%) (Fig. 4). The results of HRCT chest scan are shown in Table 4. Bilateral hilar lymphadenopathy was found in five cases (group III) where the diagnosis of sarcoidosis was ruled out based on the ocular and laboratory diagnostic criteria set by the International Ocular Sarcoidosis Workshop [11]. The bronchoalveolar biopsy was performed in only one case and was found to be negative for sarcoid.

**Table 4:** Groups of HRCT chest findings in Eales' patients suspected of pulmonary tuberculosis

Groups	Number	Percentage
I (HRCT findings typical of active TB)	1	3.4
II (HRCT findings typical of healed TB)	10	34.5
III (HRCT findings s/o sarcoidosis)	4	13.8
IV (No specific findings)	17	58.6
Total	29	100

Comparison was done between all the three tests performed to support tuberculosis in 29 Eales' patients, and Mantoux test was found to have statistically significant in comparison to chest X-ray and QuantiFERON TB Gold test (Table 5).

**Table 5:** The comparison of sensitivity and specificity among the various tests

Investigations	Sensitivity	Specificity	*p* values
Chest X-ray	66.67	46.15	0.186
Mantoux test	76.92	62.5	0.033
QuantiFERON TB Gold test	66.67	57.14	0.198

Five (17.2%) cases underwent pars plana vitrectomy for non-resolving vitreous hemorrhage and one case underwent retinal attachment surgery with encirclage. All of them have maintained stable vision during follow-up. Six patients were started on a 9-month regimen of ATT by the chest physician. These patients responded well to the treatment with complete remission of the disease at the end of 1 year. One patient developed ethambutol toxicity and subsequently developed irreversible optic atrophy despite stopping the drug. However, the Eales' patients with ATT did not have to undergo any surgical interventions compared to the Eales' patients without ATT. Less than five recurrences in the 2-year follow-up time was seen in 17 (40.4%) eyes, more than five recurrences in 9 (21.4%) eyes, and no recurrences were noted in 16 (38%) eyes. There was only one case of recurrence in the Eales' patients who was diagnosed with TB and treated with ATT. So, the recurrence among the Eales' patients with ATT was found to be lesser compared to the Eales' patients in whom ATT was not indicated but *p* value was not statistically significant.

Hence, the final visual outcome improved in 17 (40.5%) eyes and maintained in 21 (50%) eyes, but vision deteriorated in 7(16.7%) eyes. The cause of visual deterioration was long-standing inoperable retinal detachment, epiretinal membrane formation, secondary glaucoma, chronic macular edema, and optic atrophy.

Although the results of our study were limited by a small sample size, one proportion *Z* test was found to be statistically significant (*p* < 0.05) to test the significance of Mantoux test (sensitivity 76.92% and specificity 62.5%).

## Discussion

The association of Eales' disease with tuberculosis has been explored by various researchers using biochemical, histopathological, and molecular biologic studies. To the best of our knowledge, the role of HRCT chest in diagnosing tubercular etiology in Eales' disease has not been reported so far. Few studies on HRCT chest for the incidence of pulmonary TB in granulomatous uveitis have been done, but the results were not statistically significant due to the small sample size [10].

Management of Eales' diseases entails the use of systemic steroids and immunosuppressant along with several other treatment modalities. A thorough systemic evaluation to rule out active or healed tubercular infection before starting any such treatment is mandatory. We used chest X-ray, Mantoux test, QuantiFERON TB Gold, and HRCT chest to rule out the same and confirmed tuberculosis in six cases of Eales'. The recurrence rate and need for surgical intervention among Eales' patients with ATT were found to be lower compared to the Eales' patients without the need of ATT. Thus, anti-tubercular treatment can hold an important place in the management of Eales' disease amidst the existing armamentarium of treatment options such as corticosteroids (oral and periocular), immunosuppressive agents, laser photocoagulation, cryotherapy, and vitrectomy.

Mantoux test is reported to have limited specificity and sensitivity. Prior exposure to nontuberculous mycobacterium, cross reactivity with the Bacillus Calmette-Guerin (BCG) vaccine can give false positive result. In endemic country like India, Mantoux test still holds an important place, and surprisingly in our study group, the role of Mantoux test in correlating the tubercular etiology of Eales' patients was found to be significant (*p* value 0.033). This proves that even after the advancements in newer investigation modalities, Mantoux test maintains its importance in its own way.

Chest X-ray has been used as a screening tool for pulmonary TB for several years. It has a low sensitivity and negative predictive value for active primary pulmonary tuberculosis but can differentiate active from inactive disease on serial evaluations [10]. Likewise, interferon gamma release assays like QuantiFERON TB Gold is an important test but have significant limitations and cannot distinguish between latent and active tuberculosis [12]. HRCT chest can identify active disease with the help of a single scan. It detects active as well as healed lesions and also helps to differentiate between them. HRCT is better than plain chest radiography to identify the extent of pulmonary TB, especially subtle areas of consolidation, cavitation, bronchogenic, and miliary spread. The main limitations of HRCT chest are high cost and radiation exposure [13]. The post treatment HRCT chest was not repeated to optimize the cost and radiation hazard.

Though our sample size was small, this data supports the possible association of tuberculosis with Eales' disease. It also suggests HRCT chest to be a useful diagnostic test for the detection of pulmonary tuberculosis. A limitation of our study was the lack of a gold standard for comparison like real-time PCR from vitreous or other tissue samples from the eye to confirm our diagnosis, nor was organism isolation from sputum samples used, which is the standard method of diagnosis for pulmonary TB. Also, we made a presumption at the beginning of the study that the predominant site of systemic TB is the lung and extrapulmonary primary TB cases were unaccounted for. Another limitation of the study was lack of the control group to compare the results.

## Conclusions

HRCT chest is a helpful diagnostic tool in finding out tubercular lesions in the chest of Eales' patients and can provide important information regarding diagnosis and management of the Eales' disease.
